# Personalization of Repetitive Transcranial Magnetic Stimulation for the Treatment of Chronic Subjective Tinnitus

**DOI:** 10.3390/brainsci12020203

**Published:** 2022-01-31

**Authors:** Stefan Schoisswohl, Berthold Langguth, Tobias Hebel, Veronika Vielsmeier, Mohamed A. Abdelnaim, Martin Schecklmann

**Affiliations:** 1 Department of Psychiatry and Psychotherapy, University of Regensburg, 93053 Regensburg, Germany; Berthold.Langguth@medbo.de (B.L.); tobias.hebel@medbo.de (T.H.); Mohamed.Abdelnaim@medbo.de (M.A.A.); 2 Department of Psychology, Bundeswehr University Munich, 85577 Neubiberg, Germany; 3 Department of Otorhinolaryngology, University of Regensburg, 93053 Regensburg, Germany; Veronika.Vielsmeier@klinik.uni-regensburg.de

**Keywords:** repetitive transcranial magnetic stimulation, tinnitus, neuronavigation, rTMS personalization, neuromodulation

## Abstract

Background: Personalization of repetitive transcranial magnetic stimulation (rTMS) for tinnitus might be capable to overcome the heterogeneity of treatment responses. The assessment of loudness changes after short rTMS protocols in test sessions has been proposed as a strategy to identify the best protocol for the daily treatment application. However, the therapeutic advantages of this approach are currently not clear. The present study was designed to further investigate the feasibility and clinical efficacy of personalized rTMS as compared to a standardized rTMS protocol used for tinnitus. Methods: RTMS personalization was conducted via test sessions and reliable, sham-superior responses respectively short-term reductions in tinnitus loudness following active rTMS protocols (1, 10, 20 Hz, each 200 pulses) applied over the left and right temporal cortex. Twenty pulses at a frequency of 0.1 Hz served as a control condition (sham). In case of a response, patients were randomly allocated to ten treatment sessions of either personalized rTMS (2000 pulses with the site and frequency producing the most pronounced loudness reduction during test sessions) or standard rTMS (1 Hz, 2000 pulses left temporal cortex). Those participants who did not show a response during the test sessions received the standard protocol as well. Results: The study was terminated prematurely after 22 patients (instead of 50 planned) as the number of test session responders was much lower than expected (27% instead of 50%). Statistical evaluation of changes in metric tinnitus variables and treatment responses indicated only numerical, but not statistical superiority for personalized rTMS compared to standard treatment. Conclusions: The current stage of investigation does not allow for a clear conclusion about the therapeutic advantages of personalized rTMS for tinnitus based on test session responses. The feasibility of this approach is primarily limited by the low test session response rate.

## 1. Introduction

Since the early 2000s, repetitive transcranial magnetic stimulation (rTMS) has been investigated as a potential treatment option for tinnitus. These approaches were based on the concept of reducing pathological hyperactivity of the left auditory cortex via inhibitory low-frequency rTMS [1,2]. The common treatment approach in tinnitus is to stimulate the left or contra-lateral temporal or temporo-parietal cortex with up to 2000 pulses applied at 1 Hz for one or two weeks, which corresponds to ten treatment days by applying once-daily rTMS doses [3,4,5]. By the use of single sessions respectively a one-time rTMS administration with a limited number of pulses (50–200), immediate and short-term tinnitus loudness reductions can be observed [6,7,8,9]. Recent meta-analyses demonstrated an efficacy of rTMS as a treatment for chronic tinnitus [10,11,12], though results of placebo-controlled randomized clinical trials have been heterogeneous (e.g., [3,4]). While the available evidence explicitly indicates the potential of this therapeutic approach, its clinical application is hampered by heterogeneity in treatment responses [5,13] and only moderate effect sizes. Accordingly, the recommendations for rTMS as a tinnitus treatment vary across guidelines [14,15]. Consequently, several attempts have been undertaken in order to enhance the efficacy of rTMS for tinnitus, e.g., high-frequency stimulation protocols [16,17], continuous theta-burst stimulation [18] as well as prefrontal [19] or multi-site stimulation protocols [20,21,22] to name a few. Despite this large body of divergent investigations, a recent meta-analysis reported magnetic stimulations applied over the temporal cortex are still the most effective [11].

Currently, it is not clear which rTMS protocols are most appropriate for an application in tinnitus [5]. TMS effects, in general, are governed by a multitude of subject-related and rTMS-related factors as already outlined by De Ridder et al. [23]. Beyond that, tinnitus and its multifaceted manifestations with various phenotypes and etiologies potentially adds another layer of complexity to these already given interdependency of physiological and technical parameters in basic TMS investigations of the healthy brain [24,25]. These considerations fit well to findings of high inter-individual variability in rTMS treatment responses [5,13].

Considering that due to this heterogeneity in tinnitus manifestations and treatment responses for all treatment approaches—not only rTMS—there is up until now no common valid treatment for every single patient or a cure for tinnitus available [26,27]. A potential way to minimize the variability in treatment responses might be the personalization of interventions [23,28,29]. A tailored approach that is capable of adjusting intervention parameters to the necessity of the individual subject seems to constitute a promising approach to enhance the effectiveness of rTMS administration in tinnitus. Personalization of rTMS in tinnitus is possible by assessing the individual immediate responses to various stimulation protocols within so-called test sessions. The most efficient protocol can then be applied in the context of a daily treatment.

In two pilot studies, we scrutinized the validity and feasibility of rTMS test sessions in more detail. By means of several frequencies applied over different targets of the superior temporal gyrus, it was feasible to personalize rTMS via reliable sham-superior decreases of tinnitus loudness in five out of five tinnitus patients [9]. Likewise, it was possible to identify an individual rTMS protocol using the same approach via an exclusive stimulation of the temporo-parietal junction in 12 out of 22 tinnitus patients [30].

In sum, the reported findings emphasize the feasibility (reliable and sham-controlled) of rTMS test sessions demonstrating short-term tinnitus loudness reductions. However, the clinical effects of personalized rTMS in tinnitus, which means the transfer of test session results into the daily treatment scheme, have not been adequately investigated.

Only one study, namely Kreuzer et al. [31], pursued this strategy of rTMS personalization by evaluating short-term tinnitus loudness suppression following the application of short different rTMS protocols varying frequency (1 Hz, 5 Hz, 10 Hz, 20 Hz, and continuous theta-burst stimulation) and stimulation position (left and right temporo-parietal and prefrontal). In 50% of the tinnitus patients, a sham-superior response to one of the applied protocols was present throughout the test sessions. Those patients subsequently received their personalized rTMS protocol over the course of ten treatment sessions, whereas non-responders were treated with a standard protocol. Although no significant statistical differences between the personalized and the standard treatment were available, descriptive superiority as well as a higher number of treatment responders emphasize the concept of rTMS personalization as a promising way to decrease rTMS treatment variability in tinnitus [31]. Up to now, the trial described above represents the only study in this regard.

Therefore, the present investigation seeks to contribute to the branch of rTMS personalization in tinnitus with more methodological rigor in order to further evaluate the feasibility and therapeutic efficacy of rTMS personalization. Limitations of the study by Kreuzer et al. [31] were short tinnitus suppression rating periods after single sessions, no test for reproducibility of suppression effects, lack of patient randomization with brief tinnitus reduction during test sessions as well as non-navigated TMS coil placement. RTMS personalization of the present study was conducted via detailed evaluations of short-term tinnitus loudness changes over three minutes, day-to-day reliability, strict sham-superior responses to one of the verum protocols (≥10% average decrease in loudness over three minutes), and exclusive stimulations of the temporal cortex using an e-field guided neuronavigation system enabling a concise and reliable TMS coil positioning. Furthermore, the group of patients with tinnitus loudness reductions throughout test sessions was further split up by a random allocation of those into a standard and personalized treatment group. This randomization of test session responders (personalized vs. standard rTMS treatment) enables to differentiate whether the specific rTMS protocol is relevant for potential treatment effects or a positive response during the test sessions just reflects a general susceptibility to rTMS, independently from the used protocol.

Thus, the main objective of the present study was to investigate the feasibility and clinical effectiveness of personalized rTMS in contrast to the clinically most commonly used stimulation protocol as a control condition—low-frequency rTMS over the left temporal cortex.

## 2. Materials and Methods

The study at hand reports the clinical rTMS assessment of the tinnitus patient sample already described in Schoisswohl et al. [30], by means of a more stringent threshold for test session response, respectively, rTMS personalization. The trial has been registered at ClinicalTrials.gov (accessed on 10 January 2022) (NCT03957122).

### 2.1. Subjects

In order to be eligible for participation in the present study, tinnitus patients had to be between 18 and 75 years old, exhibit a tinnitus duration of more than 6 months (chronic tinnitus), and needed to be fluent in German. Further prerequisites were no presence of any serious somatic, neurological, or psychiatric condition (e.g., major depression, substance abuse, or encephalitis) as well as, if applicable, a stable medication with psychoactive drugs. Additional inclusion criteria were no present contraindications regarding TMS (e.g., known epilepsy or past epileptic seizures) and magnetic resonance imaging (MRI) (e.g., claustrophobia or metallic/electrical body implants). Parallel participation in any other tinnitus-related study or treatment was defined as an exclusion criterion.

Participants were fully informed about the objective, proceedings, and methods as well as the potential side effects of study participation and gave written informed consent prior to study onset. An applicable sample of 22 tinnitus patients (5 female) was recruited at the Interdisciplinary Tinnitus Centre Regensburg, Germany, from which N = 20 (5 female) fully participated in the present treatment study. Causes for the two dropouts during the treatment phase were tinnitus loudness increase and non-appearance at the stipulated study appointments. For analyses of test-session responses, these two treatment dropouts were not excluded.

### 2.2. Study Procedure

The present study was approved by the ethics committee of the University of Regensburg, Germany (ethical approval number: 17-820-101) and was registered at ClinicalTrials.gov (NCT03957122). The actual study start was preceded by a screening visit (week 1) consisting of eligibility determination plus informed consent, standard clinical audiometry (125 Hz–8 kHz; Madsen, Midimate, 622D, GN Otometrics, Taustrus, Denmark) as well as T1 anatomical MRI scans (MAGNETOM 1.5 Tesla, Siemens, Munich, Germany) for the purpose of neuronavigated TMS. Furthermore, several tinnitus- and health-related questionnaires had to be completed in their German versions (compare section Questionnaires and outcome measures).

Over the following two study visits (week 2), short rTMS test sessions were conducted in the attempt to identify an individual rTMS protocol per patient capable of temporarily evoking a reduction in tinnitus loudness (cf. Schoisswohl et al. [30]). The two test sessions were conducted within an interval of two days and at the same time of day (±1 h). Throughout each session, four different magnetic stimulation protocols were applied in a randomized order over the temporo-parietal junction (TPJ) on both hemispheres (compare section Repetitive transcranial magnetic stimulation). Before and after each stimulation, patients were obliged to verbally evaluate the current loudness of their tinnitus sensation at seven points in time every 30 s (three minutes) on a visual analog scale with a range from 0% (no tinnitus sensation) up to 110% (an increase in tinnitus loudness by 10%). A rating of 100% signifies no change and refers to the usual perceived level of tinnitus loudness.

Personalization of rTMS was executed by means of test session responders, defined as subjects exhibiting a mean tinnitus loudness suppression ($\bar{x}\;post-\bar{x}\;pre$) of at least 10% in the same type of verum protocol (frequency, hemisphere) on both test session days, superior to sham stimulation (suppression verum > suppression sham). In the event of multiple stimulation responses, the protocol with the strongest mean tinnitus suppression was specified as patients’ personalized rTMS protocol. If rTMS personalization was feasible, test session responders were randomly allocated to two treatment groups—personalized daily treatment (identified rTMS protocol via test session response) or standard daily treatment (1 Hz over the left TPJ) with a 50:50 chance. In case of a test session non-response, patients were automatically allocated to the standard daily treatment group. This resulted in three treatment arms: (1) test session responders with personalized daily treatment; (2) test session responders with standard daily treatment; (3) test session non-responders with standard daily treatment. We aimed for at least 12 patients in each treatment arm. By an expected number of about 50% test session responders (see Kreuzer et al. [31]) and a random allocation within the test session responders to personalized and standard treatment groups, we strove for the inclusion of 50 patients (50% test session responders = 25; 50% allocation rate to personalized or standard treatment within the test session responder group ≈ 12).

In the following two weeks (week 3 and 4), patients received ten sessions of rTMS treatment (2 × 5 working days; same daytime) accompanied by baseline and end of treatment measurements consisting of miscellaneous questionnaires (compare section Questionnaires and outcome measures). After a period of 10 weeks, a follow-up visit took place (week 14) which included the same questionnaires as during baseline and end of treatment visits.

Due to the limited number of test session responders (see results section), the study was terminated prematurely after the inclusion of 22 patients.

### 2.3. Repetitive Transcranial Magnetic Stimulation (rTMS)

RTMS sessions were executed with an e-field guided TMS machine (NBT System 2; Nexstim Plc. Helsinki, Finland) in combination with co-registered anatomical T1 brain scans allowing for visualization of strength (V/m) and direction of the induced e-field on individual 3D head models. Any stimulation was conducted with the induced e-field oriented perpendicular to the sulcus of the target brain area/gyrus of interest. Moreover, a system-integrated aiming tool allowed for a repetition of the stimulation/coil position for each applied pulse in terms of centering, rotation, and tilting. To avoid hearing damage caused by the loud TMS click noise, each patient was wearing in-ear plugs. Test sessions as well as resting motor threshold (RMT) determination followed the exact same methodological procedure as already outlined in Schoisswohl et al. [30].

Before the start of the first test session, patients’ RMT was determined for the purpose of stimulation intensity specification of test sessions and treatment sessions. Single pulses were administered at different locations over the left primary motor cortex up to the visibility of several motor evoked potentials (MEP) with a peak-to-peak amplitude of >50 µV recorded from three muscles of the right hand (musculus abductor pollicis brevis, musculus of the first dorsal interosseus, musculus abductor digiti minimi). The stimulation position which elicited the highest MEP amplitude was repeated via the system-integrated aiming tool. Next, patients’ RMT was defined by the maximum likelihood threshold hunting algorithm [32] implemented in the used TMS system.

Throughout the test sessions, 200 pulses of 1 Hz, 10 Hz, and 20 Hz rTMS served as verum magnetic stimulations, whereas 20 pulses at 0.1 Hz were deployed as a sham stimulation since this type of protocol is supposed to not provoke neuroplasticity [33,34]. All magnetic stimulation protocols were applied in a randomized order at 110% RMT over the left and right TPJ using an uncooled figure-of-eight coil (no cooling noise). In total, eight different rTMS protocols were applied per test session. Electrode positions CP5 and CP6 (10–20 system) served as a point of reference for TPJ stimulation and were marked on the structural scans via a digitization pen. Additionally, a single pulse at 10% RMT was given in order to ensure an exact replication of the coil position via the aiming tool whilst each test or treatment session.

Over the course of the subsequent treatment period, patients received 10 rTMS sessions á 2000 pulses, either with their personalized protocol or the most common clinically used rTMS protocol for tinnitus—namely left hemispheric 1 Hz (standard treatment). All treatment stimulations were conducted with an air-cooled coil at 110% RMT.

### 2.4. Questionnaires and Outcome Measures

Demographic and clinical characteristics were assessed via the European School of Interdisciplinary Tinnitus Research Screening Questionnaire (ESIT-SQ, [35]) and the Tinnitus Sample Case History Questionnaire (TSCHQ, [36]) during screening visits.

The Tinnitus Functional Index (TFI, [37]) was defined as the primary outcome for the trial (see also ClinicalTrials.gov; NCT03957122) and had to be filled out at screening, baseline, treatment end, and follow-up visits together with the following further questionnaires: the Tinnitus Handicap Inventory (THI, [38,39]), the Mini Tinnitus Questionnaire (Mini-TQ, [40]), the Major Depression Inventory (MDI, [41]), the World Health Organization—Quality of Life instrument (WHOQOL-BREF) covering the four domains physical health, psychological, social relationships, and environment [42]. Beyond that, participants had to rate the loudness of their tinnitus (0—not at all loud; 10—extremely loud), the tinnitus-induced discomfort (0—no discomfort; 10—severe discomfort), annoyance (0—not at all annoying; 10—extremely annoying), unpleasantness (0—not at all unpleasant; 10—extremely unpleasant) as well as the possibility to ignore their tinnitus (0—very easy to ignore; 10—impossible to ignore) on Visual Analog Scales (VAS). At the end of the treatment and follow-up phase, patients had to evaluate their tinnitus complaints via the Clinical Global Impression Scale for Improvement (CGI-I, [43]) compared to before treatment on a 7-point Likert Scale (1 = very much better; 2 = much better; 3 = minimally better; 4 = no change; 5 = minimally worse; 6 = much worse, and 7 = very much worse).

### 2.5. Statistical Analysis

Statistical analyses were performed with the statistic software R (R version 4.0.3; R Foundation for Statistical Computing, Vienna, Austria) using the packages “lme4”, “lmerTest”, “psych”, “sjstats”, “emmeans” and “ggplot2”. Data were analyzed by means of linear mixed-effect models separated for each assessment inventory (e.g., TFI). The following fixed effects as well as reasonable interactions were tested in each model fitting proceeding: time (screening, baseline, treatment end, follow-up), test session responder (yes/no) as well as treatment protocol (standard, personalized). Patient (id) was treated as a random effect in each model fitting proceeding. Models with the best fit for the data were derived according to Harrison et al. [44] and comparisons with likelihood ratio tests. The quantity of explained variance by the respective models was calculated by means of marginal (predictors only) and conditional (predictors and random effect) *R^2^* [45]. Fixed effects were analyzed using the expected mean square approach for each identified model. Post hoc Tukey tests were utilized to reveal possible differences within fixed effects. Effect sizes of post hoc contrasts were evaluated with Cohen’s d.

Based on the a priori defined aim of the study, potential associations of treatment group ((1) test session responder—personalized rTMS; (2) test session responder—standard rTMS; (3) test session non-responder—standard rTMS) with CGI-I ratings (condensed to the categories better, no change, worse) were analyzed using χ^2^ tests or Fisher’s exact tests in the event of cell frequencies lower than 5 separately for treatment end and follow-up visits.

Beyond that, the quantity of treatment responders (cave: not test session responders) was identified by means of two distinct approaches for the three treatment groups. First, by a 7-point decrease from baseline to treatment end in our primary outcome measure the TFI pursuant to Folmer et al. [4]; second, via a score reduction of 7 points likewise from baseline to end of treatment in the THI according to Zeman et al. [46]. Potential associations of treatment group (personalized rTMS/standard rTMS/test session responder—standard rTMS) with treatment response (yes/no) were likewise analyzed via χ^2^ tests or Fisher’s exact tests separately for the TFI and THI. The threshold for statistical significance was set at the 5% level for all analyses.

Additionally, descriptive statistics for pre- to post-treatment TFI and THI score changes (post-pre) were calculated and presented for the standard and personalized treatment groups as well as test sessions responders receiving daily standard treatment.

The average score changes in the TFI for the personalized and standard rTMS treatment groups were used for an effect size calculation (Cohen´s d) in order to deduce the needed sample size for this contrast with G*Power [47] and a significance level of 5% and a statistical power of 80% (two-tailed).

## 3. Results

### 3.1. Sample Characteristics and rTMS Side Effects

The investigated tinnitus patient sample exhibited an average age of 57.05 years (SD = 6.77), a mean tinnitus duration of 126.00 months (SD = 105.83), and the majority reported perceiving tinnitus bilateral (*n* = 13). At screening, the mean TFI and THI scores were 48.08 (SD = 17.91) and 46.80 (SD = 16.43), respectively (moderate tinnitus severity), whereas the mean Mini-TQ was 12.94 (SD = 4.28) (border between moderate and severe). No clinically relevant depression was observed in any of the tinnitus patients using the MDI (M = 14.82, SD = 9.93). Mean RMT for the treatments was 34.10% (SD = 4.70). Detailed descriptive statistics of the tinnitus sample at hand are presented in Table 1.

In addition to expected TMS-related side effects such as discomfort while stimulation or short-term increases in tinnitus loudness following stimulation, no side effects were observed over the course of test sessions. One patient canceled the rTMS treatment due to an increase in tinnitus loudness during treatment. Another patient reported a slight headache during the treatment phase. No further side effects were reported.

### 3.2. rTMS Personalization

The identification of a personalized rTMS protocol for short-term tinnitus suppression via test session response was feasible in *n* = 6 patients (27.27%). Two patients responded to 20 Hz over the left TPJ, two to 20 Hz over the right TPJ, one to 10 Hz over the left TPJ, and one to 1 Hz over the left TPJ. Based on pilot studies, we expected to have a test session responder rate of 50%. The much lower as anticipated test session responder rate led to a premature study termination as we would have to include almost twice as many patients as had been planned to randomize 25 test session responders (92 instead of 50 patients; 25/6 × 22 = 92).

On account of few test session responders, consequential study termination as well as our initial plan to randomize the group of test session responders to personalized and standard treatment groups, only *n* = 4 test session responders were subsequently treated with their personalized rTMS protocols. The other two test session responders (1 Hz right TPJ, 20 Hz left TPJ) received the standard protocol of left hemispheric 1 Hz rTMS. Both dropouts were test session non-responders and received the standard protocol for treatment.

### 3.3. Treatment Results

Linear mixed-effect model fitting identified the model *response ~ time + test session responder + treatment protocol + test session responder × treatment protocol +* (1|patient id) for the TFI, THI, WHOQOL-BREF domain 1 (physical health), and WHOQOL-BREF domain 3 (social relationships). Fixed-effect testing through the expected mean square approach revealed a significant effect of *time* for all fitted models. For the WHOQOL-BREF domain 2 (psychological) and VAS tinnitus unpleasantness, the following model with the best fit for the data could be identified: *response ~ time + test session responder + treatment protocol + time × treatment protocol + test session responder × treatment protocol +* (1|patient id). Subsequent fixed-effect testing demonstrated a significant effect of *time* for VAS tinnitus unpleasantness as well as significant interaction of *time ×* *treatment protocol* for both the WHOQOL-BREF domain 2 and VAS tinnitus unpleasantness. Detailed results of the model fitting and the fixed effect testing can be seen from Table A1 and Table A2 in the Appendix A. For all other outcome measures, no model superior to the intercept-only model could be detected.

**Figure 1:**
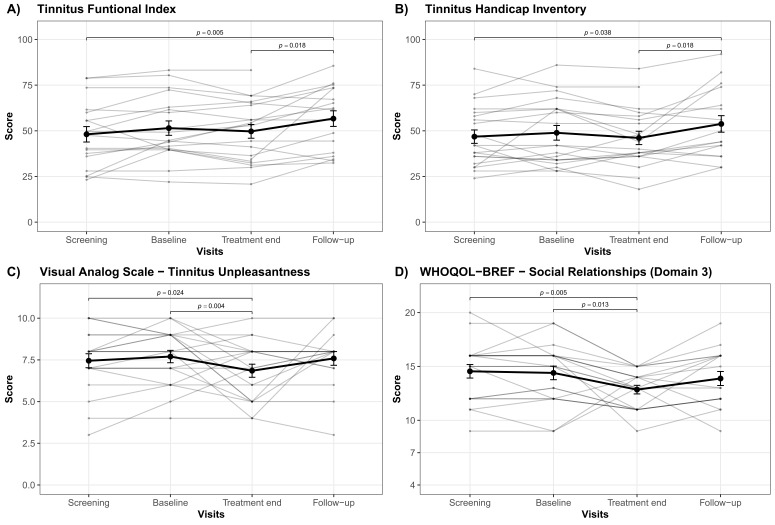
Results of post hoc analysis. Averaged total score changes over the course of all study visits (screening, baseline, treatment end, follow-up) are presented by means of bold lines for the (**A**) Tinnitus Functional Index, (**B**) Tinnitus Handicap Inventory, (**C**) Visual Analog Scale for tinnitus unpleasantness and (**D**) the social relationship domain (domain 3) of the abbreviated version of the World Health Organization Quality of Life questionnaire. Error bars indicate standard errors. Significant differences between study visits are highlighted with bars and the respective *p*-values. Greyish lines represent the total scores on a single patient level.

**Figure 2:**
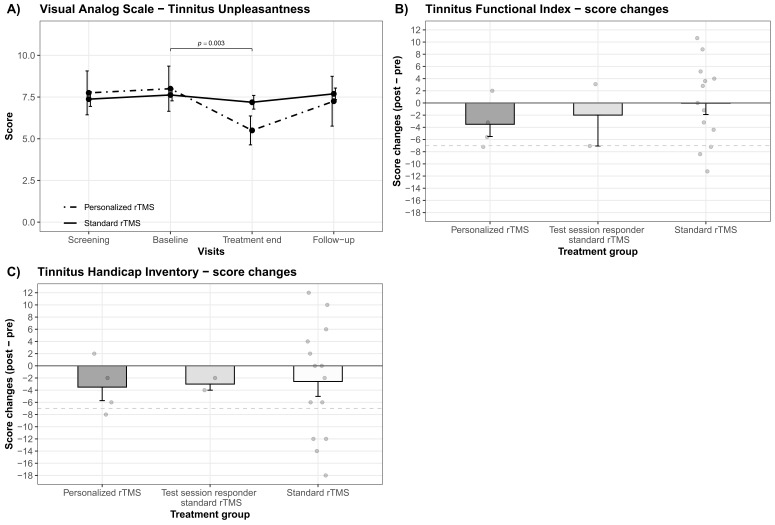
Personalized versus standard rTMS treatment. (**A**) Mean changes in the Visual Analog Scale for tinnitus unpleasantness are outlined for the treatment groups of standard and personalized rTMS for all study visits (screening, baseline, treatment end, follow-up). A significant alleviation from baseline to treatment end for the personalized rTMS treatment group is highlighted with bars and the respective *p*-value. Descriptive differences between the personalized and standard rTMS treatment group as well as test session responder receiving standard rTMS treatment are illustrated via mean score changes from baseline to end of treatment for the (**B**) Tinnitus Functional Index and (**C**) the Tinnitus Handicap Inventory. Error bars indicate standard errors. Greyish points represent mean score changes on a single patient level (TFI: 1 missing). The grey dashed line represents the cut-off for treatment response (7-point reduction).

Ensuing post hoc contrasts revealed significant differences amongst study visits for the TFI, THI, WHOQOL-BREF domain 3 (social relationships), and VAS tinnitus unpleasantness as described in the following. Significant differences between treatment end and follow-up together with significant differences between follow-up and screening have been observed for the TFI and the THI; whereby the follow-up measurements appeared to exhibit higher scores for both questionnaires (cf. Figure 1A,B). Moreover, significant differences among baseline versus treatment end as well as treatment end versus screening were present for the WHOQOL-BREF domain 3 (social relationships) and the VAS for tinnitus unpleasantness. Tinnitus unpleasantness as well as social relationships numerically decreased from screening, respectively, baseline to treatment end (cf. Figure 1C,D). A decrease in WHOQOL-BREF means a decrease in quality of life. Further, post hoc contrasts were able to detect significant differences for the VAS tinnitus unpleasantness between baseline and end of treatment exclusively for the personalized rTMS treatment group (cf. Figure 2A). Neither statistical differences at study visits, between standard and personalized treatment groups in general nor between the treatment groups at any study visit, were observed by post hoc analyses for the WHOQOL-BREF domain 1 (physical health) and domain 2 (psychological). Findings from post hoc contrasts plus relevant descriptive statistics and effect sizes are outlined in Table 2 as well as illustrated in Figure 1 and Figure 2A.

A Fisher’s exact test revealed a statistical trend for an association of treatment group with patients’ CGI-I ratings (better, no change, worse) exclusively at treatment end (*p* = 0.065). In the personalized treatment group, 2 out of 4 patients (50%) reported an improvement (1 patient—no change; 1 patient—missing), while in the standard treatment group only 2 out of 14 patients (14.29%) demonstrated an amelioration (10 patients—no change). None of the 2 test session responders, who received the standard daily treatment, indicated an improvement in the CGI-I (1 patient—worsening; 1 patient—no change).

According to our predefined treatment responder criteria, we observed a total number of *n* = 5 TFI treatment responders (25%) irrespective of the treatment group. Within the group which received their identified personalized rTMS protocol, 1 out of 4 patients (25%) was determined as a TFI treatment responder. In the standard group, 3 out of 14 patients (21.43%; 1 missing) responded to treatment with 1 Hz over the left TPJ. For the 2 test session responders who received the standard rTMS treatment, 1 patient (50%) was identified as a treatment responder using the TFI.

Responder identification via the THI revealed an identical pattern of *n* = 5 treatment responders (25%) irrespective of treatment group. One out of four (25%) patients in the personalized treatment group and 4 out of 14 (28.57%) patients in the standard treatment group were identified as treatment responders via a 7-point reduction in the THI. No treatment responders could be identified for the 2 test session responders receiving the standard treatment. Two patients were identified as responders in both approaches (test session non-response—standard treatment/test session response—personalized treatment). No statistically significant association of treatment group with treatment response was observed neither using the TFI nor the THI.

Descriptive differences between the three treatment groups revealed small but higher average score decreases from baseline to treatment end for the personalized treatment group in the TFI (personalized rTMS: M = 3.50, SD = 4.02); test session responder—standard rTMS: M = 1.99, SD = 7.18; standard rTMS: M = 0.05, SD = 6.67) as well as the THI (personalized rTMS: M = 3.50, SD = 4.43); test session responder—standard rTMS: M = 3.00, SD = 1.41; standard rTMS: M = 2.57, SD = 9.16). The standard rTMS treatment group showed the slightest changes, notably in the TFI no average score changes were observed. Descriptive score changes (post-pre) per treatment group for the TFI and THI are delineated in Figure 2B,C.

By means of average score alleviations in the TFI for the personalized and standard rTMS treatment group showing an effect size of d = 0.551, the necessary sample size to adequately contrast these two groups would be N = 106.

## 4. Discussion

The main aim of the current experiment was to demonstrate in a second study that personalized rTMS treatment is feasible and effective in tinnitus. Our initial plan was to overcome the limitations identified by Kreuzer et al. [31] and randomly allocate the group of test session responders into two arms for the subsequent treatment phase—daily personalized or standard rTMS treatment. This should have enabled us to not only control for unspecific rTMS effects but also make more valid statements about potential advantages of personalized rTMS in contrast to left temporal 1 Hz rTMS.

The preliminary study of Kreuzer and colleagues [31] showed that about half of the patients had specific single session responses and that these test session responders showed numerically superior treatment effects in the Tinnitus Questionnaire (TQ, [48]; *p* < 0.1; large effect size).

In contrast to our previous analysis (55%, [30]) as well as Kreuzer et al. (48%, [31]), the present quantity of test session responders appeared to be much lower (27%), which ended up with termination of the study ahead of schedule as we would have had to include almost twice as many patients planned (92 instead of 50), in order to appropriately allocate 25 test session responders to the two treatment groups (test session responder—personalized rTMS; test session responder—standard rTMS).

In this study, possible reasons for the given disparity in test session responses might be related to the more rigorous methodological approach. We strove for a detailed evaluation of tinnitus loudness changes (several rating points before and after short rTMS protocols) plus reliable and sham-superior responses. Moreover, we exclusively targeted the TPJ. In favor of increasing the methodological approach of rTMS personalization by more robust test session responses, we decided to use a more stringent cut-off for test session responses, respectively, rTMS personalization (≥10% average tinnitus loudness decrease). Retrospectively, this criterion might have been chosen too strictly and might be the primary reason for the low number of test session responders in the present analysis.

Despite study termination, we decided to analyze treatment data, since they have relevance for research approaches committed to the personalization of rTMS.

Only 4 out of 6 patients of the test session responder group were treated with their personalized rTMS protocol resulting in 16 patients treated with the standard protocol (2 of which were from the test session responder group). We did not observe any statistical superiority of personalized rTMS whether in our primary outcome measure (TFI) nor in any other secondary outcome measurement. Interestingly, we observed a decrease in tinnitus unpleasantness (VAS) from baseline to treatment end solely for the treatment group which received their personalized rTMS protocol (cf. Figure 2A). Moreover, an improvement in the CGI-I tended to be associated with the personalized rTMS treatment. A descriptive comparison of the three treatment groups indicated a small but superior tinnitus distress alleviation from pre to post treatment for patients who received their personalized rTMS protocol (cf. Figure 2A,B). Even if these results are in line with Kreuzer et al. [31], they should not be overinterpreted as they come from only a few patients and are only found in some (secondary) outcome measurements.

In addition, we used the data from the present study for sample size estimation. For the contrast personalized vs. standard rTMS treatment, a sample size of N = 106 would be needed, which is more than twice as much as our aspired investigation of 50 tinnitus patients. As only 6 out of 22 investigated patients demonstrated a response in the test session, one would have needed a sample of several hundred patients for a sufficiently powered study.

According to our predefined treatment responder criteria, 25% of patients responded to a daily treatment with their personalized rTMS protocol using the TFI and THI. While in the group of test session non-responders treated with the standard rTMS protocol, 21% (TFI) respectively 29% (THI) were identified as treatment responders. One of the two test session responders who received standard rTMS treatment was identified as a treatment responder. These findings are in contrast to the results of Kreuzer et al. [31], who not only reported a higher overall treatment responder rate using a sample of almost the same size but also a higher number of treatment responders in the group of personalized (58%) in contrast to a standard treatment group (42%) by means of a 5-point reduction in the Tinnitus Questionnaire [48].

Possible reasons for disparities in treatment responses between Kreuzer et al. [31] and the present study might be differences in the applied treatment (dual-site vs. single-site rTMS) and in the used outcome variables (TQ and tinnitus loudness vs. several others). Unlike the study at hand, Kreuzer et al. [31] applied a multisite stimulation protocol with 20 Hz over the left dorsolateral prefrontal cortex followed by 1 Hz over the left temporal cortex, respectively, both temporal cortices as a standard rTMS protocol. Since tinnitus-related activity changes were also reported for frontal regions of the cortex [49,50,51,52] and trials were able to report positive effects of prefrontal rTMS [19,53], the inclusion of prefrontal stimulation targets might reduce inter-subject variability in rTMS responses resulting in a higher number of test sessions and treatment responders. Likewise, it has been shown that rTMS applied over multiple regions appears to be superior to a single-site stimulation [21,54,55,56]. However, there is also research suggesting that magnetic stimulation of the temporal cortex seems to be the most efficacious rTMS protocol [11], leaving open the question regarding the superiority of multi-site rTMS.

In the current experiment, we used a 7-point reduction in the TFI [4] as treatment responder criterion. Besides the appropriateness of the TFI for research purposes [57], a global score reduction of 22.4 points [58] respectively 13 points [37] is suggested as a minimal clinically important difference. Adhering to these thresholds, none of our investigated tinnitus patients would be designated as a treatment responder, indicating rather small clinical responses in the current sample, which further hampers the clinical applicability.

Combining the insights gained from both studies on rTMS personalization in tinnitus so far, personalization of study protocols based on the effect of test sessions is only feasible in a rather small subgroup of tinnitus patients. Descriptive results suggest a potential superiority of personalized protocols, but the effect size seems to be too small to reach clinical relevance. Despite the lack of a clear statement at the current stage of investigation, it should not be concluded from the present data, that personalization of rTMS protocols does not make any sense. Test session protocols as well as outcome parameters might have been chosen suboptimal in the present study. It remains to be tested, whether other protocols involving priming, multi-site, or theta-burst stimulation might be more appropriate and whether neurophysiological readout parameters (e.g., EEG) represent more suitable response criteria. Challenges for the future are a careful selection of stimulation parameters for test sessions in light of practicability or time-intensiveness as with, e.g., different stimulation positions, stimulation intensities, and putative protocols numerous test session options are possible.

In terms of general rTMS efficacy, we merely observed a descriptive decrease in tinnitus distress from pre to post treatment in the TFI and THI (Figure 1A,B). No clinically relevant effect, more specifically no significant amelioration of tinnitus distress in contrast to before rTMS treatment, could be demonstrated. These findings further question the usefulness of neuronavigated 1 Hz rTMS treatment applied over the left TPJ, as this protocol was applied as standard treatment in the current study.

Expectations of patients might have been higher in the present study than in former investigations of our work group as we explicitly aimed for reductions in tinnitus loudness. In previous studies, patients were rather informed about the general benefits of rTMS. Being a participant experiencing only minor to no loudness changes during the test sessions, consequently receiving the standard protocol for the treatment phase, might have resulted in disappointment and thus might have induced nocebo-like effects.

In the absence of any significant improvement in clinical measures of tinnitus severity, we observed a significant reduction in tinnitus unpleasantness after ten sessions of rTMS in contrast to screening and baseline assessments (Figure 1C). A similar pattern was observed in a study using ten sessions of transcranial random noise stimulation. Even though tinnitus distress increased, tinnitus-related unpleasantness decreased compared to treatment starting on a descriptive level [59]. However, other rTMS studies report significant effects on tinnitus distress along with no effects on tinnitus-related unpleasantness [18,55,60]. Considering that together with the absence of an effect in any other outcome measure, this finding should only be interpreted with caution. Interestingly, we also observed a reduction in social relationships from the initial screening visit to treatment end as well as during the treatment phase (Figure 1D). Since patients might focus more on their tinnitus percept than usual, already starting from the first assessment onwards, a more intense occupation could lead to more social isolation in some tinnitus patients. Missing social support might further result in higher distress in some individuals and potentially influence TFI and THI scores.

In our preceding analysis, we opted for an identification of rTMS test session responders based on reliable and sham-superior increases in the alpha respectively decreases in the gamma frequency band [30] based on prevalent neurophysiological models in tinnitus [61,62]. Future studies should strive for a sophisticated analysis of EEG activity changes before and after rTMS treatments to identify potential electrophysiological biomarkers which could then be targeted during test sessions. According to a recent study, response to a rather short rTMS treatment is linked to a power reduction in the gamma frequency band as well as enhanced coherence in the beta frequency range [63].

Due to the small sample size, we refrained from the inclusion of other demographic variables in our model-fitting approach as well as comparisons of demographic differences between treatment groups. Besides laterality of hearing loss [64], no predictor for rTMS treatment response is currently available [65].

In view of the present findings and insights, future studies with lower test session response thresholds for rTMS treatment personalization, additional stimulation positions next to temporal targets, electrophysiological investigations before and after treatment as well as larger sample sizes allowing for the proper distribution of treatment groups are highly needed at this stage of research concerning rTMS personalization in the field of tinnitus.

## 5. Conclusions

In the present study, we wanted to investigate the effectiveness of personalized rTMS in contrast to the most frequently used rTMS protocol—1 Hz over the left TPJ. By virtue of a low number of test session responders and the accompanying unbalanced treatment groups, the study was prematurely terminated. The present findings indicate that only a rather small subgroup of all patients demonstrated a response during the test sessions and that in these patients the personalized protocol seems to be at best marginally superior to standard daily treatment. Considering current investigations, no conclusive statement about the therapeutic advantages of personalized rTMS for tinnitus can be deduced at this early stage of the investigation.

## Figures and Tables

**Table 1:** Sample characteristics.

N (female)	20 (5)			
Handedness (left/right/both) (3 missings)	0/13/4			
Tinnitus laterality (left/right/both/inside head) (3 missing)	1/1/13/2			
	**M ± SD**	**Md**	**Min**	**Max**
Age (years)	57.05 ± 6.77	57.50	43.00	69.00
Tinnitus duration (months) (2 missing)	126.00 ± 105.83	102.00	14.00	420.00
Hearing loss left (dB) (7 missing)	23.60 ± 10.10	22.22	7.22	41.67
Hearing loss right (dB) (7 missing)	28.39 ± 14.74	23.89	7.78	61.86
RMT (%)	34.10 ± 4.70	33.50	27.00	44.00
TFI score (0–100) (2 missing)	48.08 ± 17.91	48.55	23.20	78.80
THI score (0–100)	46.80 ± 16.43	42.00	24.00	84.00
Mini-TQ score (0–24) (3 missing)	12.94 ± 4.28	13.00	7.00	20.00
MDI score (0–50) (3 missing)	14.82 ± 9.93	14.00	1.00	40.00
VAS tinnitus loudness (0–10)	7.15 ± 1.69	7.50	3.00	10.00
VAS tinnitus discomfort (0–10)	7.50 ± 1.61	8.00	4.00	10.00
VAS tinnitus annoyance (0–10)	6.60 ± 2.30	7.00	2.00	10.00
VAS tinnitus ignorability (0–10)	7.60 ± 2.14	8.00	3.00	10.00
VAS tinnitus unpleasantness 0–10)	7.45 ± 1.88	8.00	3.00	10.00
WHOQOL-BREF domain 1 (Physical health) (4–20)	12.35 ± 2.01	13.00	8.00	15.00
WHOQOL-BREF domain 2 (Psychological health) (4–20)	13.80 ± 2.19	14.00	10.00	18.00
WHOQOL-BREF domain 3 (Social relationships) (4–20)	14.55 ± 2.80	15.50	9.00	20.00
WHOQOL-BREF domain 4 (Environment) (4–20)	16.45 ± 1.99	16.50	13.00	19.00

M = mean; SD = standard deviation; Md = Median; Min = minimum; Max = maximum; RMT = resting motor threshold; TFI = Tinnitus Functional Index; THI = Tinnitus Handicap Inventory; Mini-TQ = Mini Tinnitus Questionnaire; MDI = Major Depression Inventory; VAS = Visual Analog Scale; WHOQOL-BREF = World Health Organization Quality of Life—abbreviated.

**Table 2:** Post hoc Tukey contrasts.

Contrast	M ± SD	Estimate	T _(df, se)_	*p*	d
**TFI**					
Treatment end—follow-up	49.69 ± 16.80—56.68 ± 17.62	−7.65	−3.05 _(57.40, 2.51)_	0.018	0.406
Follow-up—screening	56.69 ± 17.62—48.08 ± 17.91	9.14	3.50 _(57.70, 2.61)_	0.005	0.484
**THI**					
Treatment end—follow-up	46.10 ± 16.31—53.76 ± 18.68	−7.69	−3.04 _(60.40, 2.53)_	0.018	0.437
Follow-up—screening	53.76 ± 18.68—46.80 ± 16.43	6.99	2.76 _(60.40, 2.53)_	0.037	0.396
**WHOQOL-BREF domain 3**					
Baseline—treatment end	14.40 ± 2.82—12.85 ± 1.79	1.55	3.14 _(60.20, 0.49)_	0.014	0.656
Treatment end—screening	12.85 ± 1.79—14.55 ± 2.80	−1.70	−3.45 _(60.20, 0.49)_	0.005	0.702
**VAS—Tinnitus unpleasantness**					
Baseline—treatment end	7.70 ± 1.66—6.85 ± 1.76	1.47	3.50 _(63.70, 0.42)_	0.004	0.497
Treatment end—screening	6.85 ± 1.76—7.45 ± 1.88	−1.22	−2.91 _(63.70, 0.42)_	0.025	0.329
**Personalized rTMS**					
Baseline—treatment end	8.00 ± 2.71—5.50 ± 1.73	2.50	3.33 _(63.70, 0.75)_	0.003	1.100

TFI = Tinnitus Functional Index; THI = Tinnitus Handicap Inventory; WHOQOL-BREF domain 3 = World Health Organization Quality of Life—abbreviated—domain 3 (social relationships); VAS = Visual Analog Scale; _df_ = degrees of freedom; _se_ = standard error; d = Cohen’s d.

## Data Availability

Not applicable.

## Appendix A

**Table A1:** Model fitting.

Model	R^2^ (Marginal)	R^2^ (Conditional)	df	AIC	BIC	logLIK	LRT	*p*
**TFI**								
Intercept only: response ~1 + (1|id)	0	0.78		579.83	586.74	−286.91		
Fitted model: response ~ time + test session responder + treatment protocol + test session responder × treatment protocol + (1|id)	0.13	0.82	5	574.42	592.85	−279.21	15.41	0.009
**THI**								
Intercept only: response ~1 + (1|id)	0	0.79		600.27	607.30	−297.13		
Fitted model: response ~ time + test session responder + treatment protocol + test session responder × treatment protocol + (1|id)	0.14	0.82	5	597.08	615.83	−290.54	13.19	0.022
**WHOQOL-BREF domain 1**								
Intercept only: response ~ 1 + (1|id)	0	0.87		240.88	247.92	−117.44		
Fitted model: response ~ time + test session responder + treatment protocol + test session responder × treatment protocol + (1|id)	0.14	0.88	5	240.26	259.01	112.13	10.62	0.059
**WHOQOL-BREF domain 2**								
Intercept only: response ~1 + (1|id)	0	0.83		265.71	272.74	−129.85		
Fitted model: response ~ time + test session responder + treatment protocol + time × treatment protocol + test session responder × treatment protocol + (1|id)	0.10	0.86	8	265.86	291.64	−121.93	15.86	0.045
**WHOQOL-BREF domain 3**								
Intercept only: response ~1 + (1|id)	0	0.57	5	342.61	349.64	−168.30		
Fitted model: response ~ time + test session responder + treatment protocol + test session responder × treatment protocol + (1|id)	0.20	0.65		334.56	353.31	−159.28	18.05	0.003
**VAS tinnitus unpleasantness**								
Intercept only: response ~1 + (1|id)	0	0.58		281.04	288.07	−137.52		
Fitted model: response ~ time + test session responder + treatment protocol + time × treatment protocol + test session responder × treatment protocol + (1|id)	0.08	0.67	8	282.08	307.86	−130.04	14.96	0.059

TFI = Tinnitus Functional Index; THI = Tinnitus Handicap Inventory; WHOQOL-BREF domain 1 = World Health Organization Quality of Life—abbreviated—physical health; WHOQOL-BREF domain 2 = World Health Organization Quality of Life—abbreviated—psychological; WHOQOL-BREF domain 3 = World Health Organization Quality of Life—abbreviated—social relationships; VAS = Visual Analog Scale; df = degrees of freedom; AIC = Akaike Information Criterion; BIC = Bayesian Information Criterion; logLik = log-likelihood; LRT = Likelihood Ratio Test.

**Table A2:** Fixed effect testing.

	numDF	denDF	F	*p*
**TFI**				
Time	3	54.31	5.01	0.004
**THI**				
Time	3	57.01	3.85	0.014
**WHOQOL-BREF domain 1**				
Time	3	57.11	2.78	0.049
**WHOQOL-BREF domain 2**				
Time × treatment protocol	3	57.05	3.89	0.013
**WHOQOL-BREF domain 3**				
Time	3	57.35	5.20	0.003
**VAS tinnitus unpleasantness**				
Time	3	57.01	5.29	0.003
Time × treatment protocol	3	57.01	3.00	0.038

TFI = Tinnitus Functional Index; THI = Tinnitus Handicap Inventory; WHOQOL-BREF domain 1 = World Health Organization Quality of Life—abbreviated—physical health; WHOQOL-BREF domain 2 = World Health Organization Quality of Life—abbreviated—psychological; WHOQOL-BREF domain 3 = World Health Organization Quality of Life—abbreviated—social relationships; VAS = Visual Analog Scale; numDF = degrees of freedom numerator; denDF = degrees of freedom denominator.
